# Proteomic Insight into the Symbiotic Relationship of *Pinus massoniana* Lamb and *Suillus luteus* towards Developing Al-Stress Resistance

**DOI:** 10.3390/life11020177

**Published:** 2021-02-23

**Authors:** Haiyan Liu, Houying Chen, Guijie Ding, Kuaifen Li, Yao Wang

**Affiliations:** 1 Institute for Forest Resources & Environment of Guizhou, Guiyang 550025, China; liuhaiyan301@163.com (H.L.); chenhy921934@163.com (H.C.); likuaifengzu@126.com (K.L.); 2 Guizhou Botanical Garden, Guiyang 550004, China; gzszwywy@163.com

**Keywords:** *Pinus massoniana*, aluminum stress, ectomycorrhizal fungi, differential expressed proteins

## Abstract

Global warming significantly impacts forest range areas by increasing soil acidification or aluminum toxicity. Aluminum (Al) toxicity retards plant growth by inhibiting the root development process, hindering water uptake, and limiting the bioavailability of other essential micronutrients. *Pinus massoniana* (masson pine), globally recognized as a reforestation plant, is resistant to stress conditions including biotic and abiotic stresses. This resistance is linked to the symbiotic relationship with diverse ectomycorrhizal fungal species. In the present study, we investigated the genetic regulators as expressed proteins, conferring a symbiotic relationship between Al-stress resistance and *Suillus luteus* in masson pine. Multi-treatment trials resulted in the identification of 12 core Al-stress responsive proteins conserved between Al stress conditions with or without *S. luteus* inoculation. These proteins are involved in chaperonin CPN60-2, protein refolding and ATP-binding, Cu-Zn-superoxide dismutase precursor, oxidation-reduction process, and metal ion binding, phosphoglycerate kinase 1, glycolytic process, and metabolic process. Furthermore, 198 Al responsive proteins were identified specifically under *S. luteus*-inoculation and are involved in gene regulation, metabolic process, oxidation-reduction process, hydrolase activity, and peptide activity. Chlorophyll a-b binding protein, endoglucanase, putative spermidine synthase, NADH dehydrogenase, and glutathione-S-transferase were found with a significant positive expression under a combined Al and *S. luteus* treatment, further supported by the up-regulation of their corresponding genes. This study provides a theoretical foundation for exploiting the regulatory role of ectomycorrhizal inoculation and associated genetic changes in resistance against Al stress in masson pine.

## 1. Introduction

*Pinus massoniana* Lamb., also known as masson pine, is native to South China and a pioneer species in the forest chain dominating the subtropical areas of East Asia [1]. Salient features, including resistance to environmental stress, ability to grow in marginal soils, and tolerance to metal contaminated soils, have enabled this species to be highly used for afforestation and reforestation in China [2,3]. *P. massoniana* accounts for 7.74% of the total arborical forest zone in China [4]. Masson pine has been widely adopted for its timber and pulp due to the fast-growing habit and the high yield advantages [5]. Besides its economic importance, the afforestation of this species has potentially contributed to improve the ecosystem productivity and carbon sequestration [1].

*Pinus* spp. have been widely used in different reforestation programs worldwide [6]. Their dependency on symbiosis, hosting a wide range of ectomycorrhizal fungal species [7,8,9], is advantageous for their optimal growth and development under various natural environmental conditions [10]. The symbiotic effect of ectomycorrhizal fungi is well known for improving the growth of the host plants [9] with enhanced tolerance towards environmental stresses [11,12,13,14]. Studies have shown that *Pinus* species inoculated with ectomycorrhizal fungi display improved photosynthesis, water uptake, nutrient utilization, and immune system [11,12].

The continuous decline in forest population is mainly attributed to soil acidification, resulting from air pollution and intensive fertilizer applications. Moreover, nutrient depletion coupled with an accumulation of toxic elements also causes adverse effects on the forest population. The acidification of soils associated to an increased level of Aluminum (Al), causes a substantial reduction in plant growth by inhibiting root development [15,16], water uptake [17,18], and translocation of nutrients [19,20]. A controlled inoculation of ectomycorrhizal fungi is a useful approach for enhancing the performance of out-planted seedlings [7,21]. Previous reports showed enhanced fitness in different *Pinus* species against toxic concentrations of heavy metals under mycorrhizal fungus inoculation [22,23,24,25,26]. *Suillus luteus,* an ectomycorrhizal fungus, is considered a symbiotic solution for heavy-metal toxicities, including Al^3+^ stress [27,28,29,30]. Another study described the positive growth regulation of *P. massoniana* under the inoculation of the mycorrhizal fungus, *Pisolithus tinctorius* [31].

Morphophysiological changes and cellular responses under Al-stress conditions are ascribed to the gene expression and cellular metabolism. Previous reports for Al stress resistance in different plants identified multiple pathways including, membrane transporters, oxidative stress pathways, primary metabolism, cell wall synthesis, and protein metabolism [32,33,34,35,36,37,38,39,40,41,42,43]. However, the genetic pathways for induced Al stress tolerance in *P. massoniana* are not well documented. In particular, clarifying the contribution of ectomycorrhizal fungi in *P. massoniana* resistance to abiotic stresses has been the focus of recent studies [44,45]

The goal of this study was to identify the key proteins underlying the alleviated Al toxicity in *P. massoniana* under ectomycorrhizal fungi inoculation. We employed a comparative proteomics approach to probe the symbiotic relationship of *S. luteus* and *P. massoniana* seedlings in response to Al toxicity. This study provides insight into the proteome variations induced by ectomycorrhizal fungi under Al toxicity in *P. massoniana*.

## 2. Materials and Methods

### 2.1. Plant Material and Ectomycorrhizal Fungus

The investigated tree species in this study was *P. massoniana.* The seeds were collected from the tree Huang 12 located in Duyun City, Guizhou Province, China. The ectomycorrhizal fungus species *Suillus luteus* (SL) was used for plant inoculation. The fruiting body was collected from the pine forest of Longli Forest Farm, Longli County, Guiyang City, Guizhou Province. The inoculum was prepared, followed by inoculation, according to the methods described by Yu et al. [44]. The seedlings inoculated with *S. luteus* were selected after six months of germination, and the control plants were kept without inoculation of SL.

The research was conducted in a greenhouse with a sand culture and parameters (light intensity of 600–800 μmol m^−2^s^−1^, relative humidity of 55%, photoperiod of 16 h, 25 °C, and 18 °C in the dark) were kept constant during the growth period. For sand culture, quartz-sand purchased from Kasper Building Materials Company (Potter, WI, USA) was used. Thoroughly rinsed quartz-sand was sterilized and then filled into nutrition bowls. Selected seedlings with consistent growth were transplanted into pots. Al stress treatment was applied to the selected seedlings after normal growth for two weeks [45].

### 2.2. Collection, Separation, and Purification of Suillus Luteus

The fruit-bodies of *S. luteus* were searched in the masson pine forest by trampling. The collected fruit-bodies were cleaned to get rid of any surface contamination. The cleaned fruit-bodies were individually numbered and placed in a Ziplock bag, placed in an icebox, and brought back to the laboratory. Young and tender fruit-bodies were selected for further experiment. Separation work was carried out on an ultra-clean workbench. The surface of the fruit-bodies was disinfected with 75% alcohol and rinse twice with sterile water. The fruit-bodies were then transferred to Pachlewski medium and placed in an incubator for dark culture at 25 °C with a 50% humidity level. Hyphae were collected after appearance and cultured in a new medium until the strain was purified [46]. To confirm the purity, mycelium was collected from the outer surface and transferred to the Pachlewski medium. Active mycelium was collected and transferred from the solid cultured colony to the Pachlewski medium and observed for ten days of cultivation. The samples with no contamination were stored in the refrigerator at 4 °C for later use [47].

### 2.3. Identification of Suillus Luteus Strain

The purified fungus was inoculated in Pachlewski medium and cultivated for 15 days. According to Fan Yongjun’s method, DNA extraction was performed, followed by PCR-based amplification. The amplified DNA was sequenced, and the ITS sequence was cut and uploaded to NCBI, and the Blastn comparison was performed in the NCBI database. The fungal strain was confirmed with 99% similarity, as formerly elaborated by Du et al. [48] and Feng et al. [49].

### 2.4. Stress Treatments

The test set included 4 treatments, namely 2 inoculated LB-0 (Al^3+^, 0 mmol L^−1^), LB-04 (Al^3+^, 0.4 mmol L^−1^) and two non-inoculated treatments CK-0 (Al^3+^, 0 mmol L^−1^), CK-04 (Al^3+^, 0.4 mmol L^−1^. AlCl_3_ (Tianjin Komiou Chemical Reagent Co., Ltd.) was added to Hoagland solution (complete nutrient solution), and pH of the Hoagland solution was adjusted to 4.1 ± 0.1 by adding 0.1 M diluted HCl or NaOH. The nutrient composition of Hoagland solution was as 5 mmol L^−1^ KNO_3_, 4.5 mmol L^−1^ Ca(NO_3_)·4H_2_O, 2 mmol L^−1^ MgSO_4_·7H_2_O, 1 mmol L^−1^ KH_2_PO_4_, and 25 μmol L^−1^ Fe-Na EDTA [50]. To maintain the quality, the treatment solution was changed after a week interval. Al activity was maintained by adding CaCl_2_ solution purchased from Xilong Chemical Co., Ltd., into the nutrient solution, a standard treatment acting as a buffer to avoid possible interface between Al ions and solution ions [51]. Due to the unstable Al ions, the high concentration of phosphorus in the nutrient solution can cause a reaction with Al ions and reduce the actual Al concentration of the treatment. Hence, after adding a low concentration of CaCl_2_, Ca^2+^ can preempt the binding site of Al^3+^, which ensures the target Al^3+^ concentration [52,53]. The proteome was determined from the needle samples collected 60 days after Al treatment.

### 2.5. Protein Extraction

The needles and samples of the same parts of *P. massoniana* seedlings with different treatments were selected for protein extraction by utilizing the phenol extraction method [54]. Tissue samples of pinus needles (stored at −80 °C before use) were weighed and ground into a fine powder. Phenol extraction buffer was added to the powdered samples, followed by sonication. The samples were centrifuged at 5500xg for ten minutes after adding Tris-balanced phenol. After centrifugation, methanol was added to supernatant and kept overnight. 8M urea was added to the overnight-kept samples to reconstitute the pallet. Later, protein concentration was estimated for each sample with a Bio-Rad protein assay kit (BCA kit, Bio-Rad, Hercules, CA, USA).

### 2.6. LC-MS/MS Analysis 

To perform Liquid Chromatography with tandem mass spectrometry, HPLC was used to fractionate the tryptic peptides before mass spectrometry analysis. The parameters used for the step gradient were set to acetonitrile (pH 9.0); 8–32%. Later, we combined the peptides, followed by freeze-drying in a vacuum chamber. EASY-nLC 1200 UPLC system (Thermo Fisher Scientific, Waltham, MA, USA) was employed to separate dissolved peptides. The liquid phase gradient was set to B (Methanole %); 9–25% for 30 min; 25–35% for 22 min; 35–80% for 4 min; 80% for 4 min, while the flow rate was kept constant at 350 nL/min. Orbitrap Fusion LUMOS platform was utilized to perform Tandem mass spectrometry (MS/MS) with standard mass spectrometry parameters.

The MS raw data for each sample were searched using the MASCOT engine (Matrix Science, London, UK; version 2.2) embedded into Proteome Discoverer 1.4 software for the identification and quantitation analysis. Peak lists were searched against Green Plants (*Viridiplantae*) database in Oligo 7 using the following parameters: enzyme, trypsin; maximum missed cleavage, 2; fixed modification, carbamidomethylating (C); variable modification, oxidation (M) and TMT (protein N-terminus and K); mass tolerance at 20 ppm; MS/MS mass tolerance at 0.1 Da; false discovery rate (FDR) < 0.01. Significance was assessed by ratios of TMT reporter ion intensities in the MS/MS spectra.

### 2.7. Functional Classification of Proteins

The proteins were annotated to Gene Ontology (GO) using blast2go (https://www.blast2go.com (accessed on 12 January 2021)). Protein IDs were transformed to UniProt IDs to match the corresponding GO IDs, and the relative information was obtained. IDs with no information from UniProt were subjected to InterProScan to obtain predicted GO functions of the proteins. Fisher’s exact two-terminal test with a significance threshold of *p*  <  0.05 was used to test the proteins. 

### 2.8. Protein Quantification and Differential Expressed Protein Analysis

For protein quantification, the proteins containing at least two unique peptides were enumerated for all labeled samples. The quantifiable proteins were listed first, and the abundance ratio (treated/control) was log2 transformed. The differentially expressed proteins (DEPs) between treatments were identified with fold change > 1.2 and *p* < 0.05, FDR < 0.01.

### 2.9. Expression Profile of Related Genes based on qRT-PCR

Expression profiles of genes governing differentially expressed proteins were estimated using Real-Time Quantitative Reverse Transcription PCR (qRT-PCR). Genes related to Al-responsive proteins were identified and selected, and specific primers for qRT-PCR, corresponding to the identified genes, were designed using Oligo 7 software (https://www.oligo.net/ (accessed on 12 January 2021)) (Table 1) before qRT-PCR. The primers of 9 selected genes were synthesized by Sangon Biotech (Shanghai, China). RNA was extracted from masson pine needles using Tiangen RNAprep Pure Plant kit (Tiangen Biotech, Beijing, China). A list of primers for the nine genes is presented in Table 1. The 2−^△△Ct^ method was used to calculate the relative expression levels [55].

## 3. Results

### 3.1. Overview of the Proteome Profiling in Masson Pine Needle Samples

Proteome profiling resulted in 79,046 spectra, 18,737 peptides, 16,670 unique peptides, and 4312 proteins being identified in masson pine. The detailed information on mass spectrometry collection and identification are shown in Table S1. Most of the peptides were comprised of 7–20 amino acids, which is within the ranges of proteome quality requirements [54] (Figure S1).

### 3.2. Differentially Expressed Proteins Analysis and Functional Annotations

**Figure 1:**
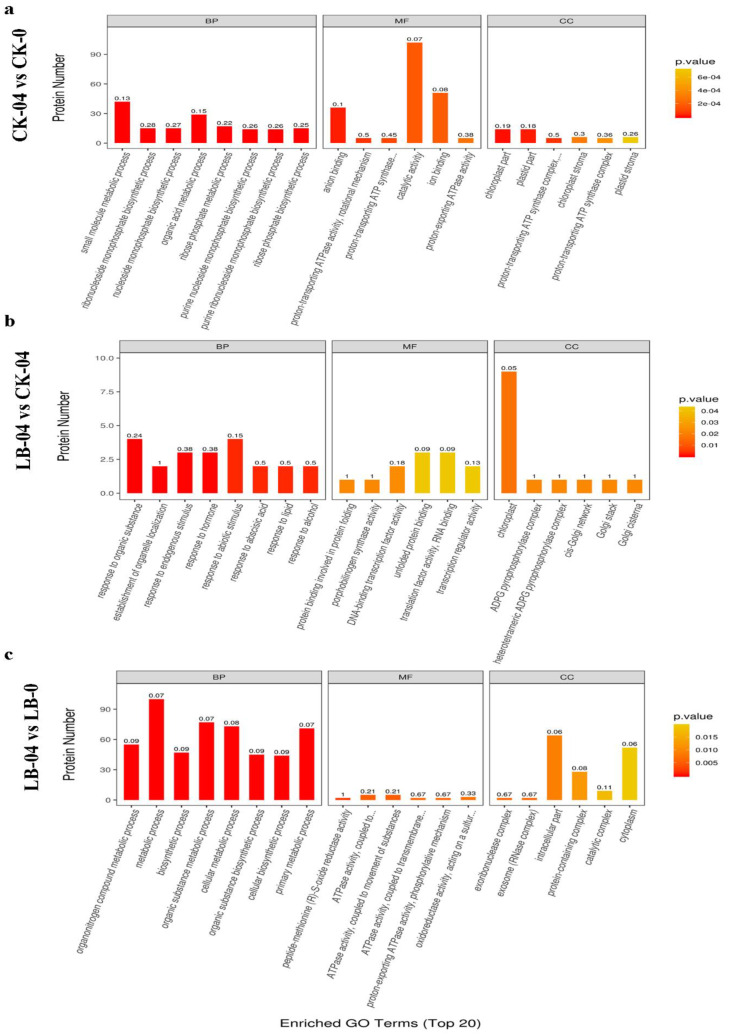
Gene ontology enrichment analysis for (**a**) LB-0 vs. CK-0 (**b**) LB-04 vs. CK-04 (**c**) LB-04 vs. CK-0. BP = Biological processes MF = molecular Functions CC = cellular components. The number on each column represents the corresponding rich factor.

The differentially-expressed proteins (DEPs) were screened by a standard screening criterion [54]: fold change > 1.2-fold (up-regulated by more than 1.2-fold or down-regulated by less than 0.83-fold) and p-value < 0.05. The comparison of CK-04 vs. CK-0 group (effect of Al stress on masson pine seedlings without inoculation) showed 215 DEPs, including 137 up-regulated and 78 down-regulated proteins in CK-04 (Table S2). These DEPs were subjected to a gene ontology (GO) enrichment analysis. The GO enrichment (Figure 1a) showed that these DEPs were mainly involved in small molecule metabolic process, catalytic activity, and ion binding. The functions of these proteins were identified as catalytic activity, binding, structural molecule activity, transporter activity, and antioxidant activity.

Under Al-stress condition, the comparison of LB-04 vs. CK-04 groups (combined effects of Al stress and *S. luteus* inoculation on masson pine seedlings) showed 96 DEPs with 50 up-regulated and 46 down-regulated proteins in LB-04 (Table S3). GO annotation (Figure 1b) for these DEPs revealed that the proteins were associated with response to an organic substance, response to abiotic stimulus, and unfolded protein binding.

Similarly, when comparing LB-04 vs. LB-0 groups (effect of Al stress on *S. luteus*-inoculated masson pine seedlings), we identified 210 DEPs, including 82 up-regulated and 128 down-regulated proteins in LB-04 (Table S4). GO annotations (Figure 1c) identified the main functions of these DEPs as metabolic process, organic substance metabolic process, cellular metabolic process, and primary metabolic process. These DEPs were involved in response to a stimulus, cellular component organization or biology, and localization.

### 3.3. Identification and Analysis of the Core Al Responsive Proteins 

**Figure 2:**
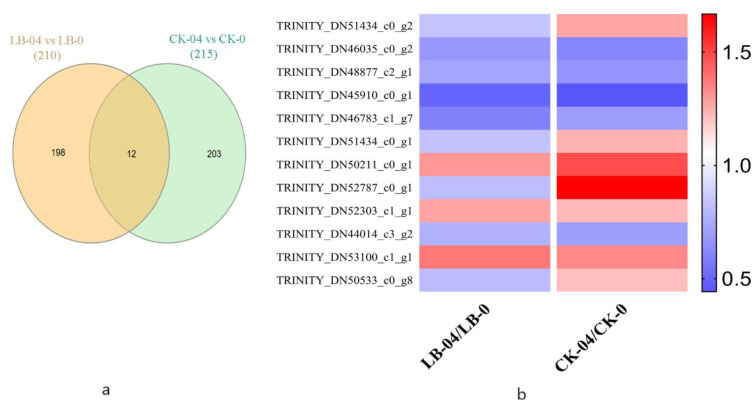
(**a**) Venn diagram representing differentially expressed proteins conserved between LB-04 vs. LB-0 and CK-04 vs. CK-0, (**b**) Differential expression pattern of proteins conserved between LB-04 vs. LB-0 & CK-04 vs. CK-0. Deep blue color shows lower intensity, and deep red color displays higher intensity. Different samples (LB-04/LB-0 and CK-04/CK-0) are represented as columns.

The core Al responsive proteins were identified by comparing DEPs between Al stress and control conditions, independently of SL-inoculation. These comparisons include LB-04 vs. LB-0 and CK-04 vs. CK-0. Venn diagram analysis revealed only 12 conserved DEPs among the two groups (Figure 2 and Table S5). This indicates that the DEPs in LB-04 vs. LB-0 group were different from CK-04 vs. CK-0 group, depicting a differential pattern of expression under the inoculation of *S. luteus*.

Among the core Al responsive proteins, three proteins, *TRINITY_DN51434_c0_g1*; chaperonin CPN60-2, *TRINITY_DN52787_c0_g1*; phosphoglycerate kinase 1, and *TRINITY-DN50533-c0-g8*; unknown protein, were up-regulated under CK-04 vs. CK-0, while their expression was down-regulated in LB-04 vs. LB-0. The other proteins showed a similar regulation pattern in both groups, demonstrating the conserved roles of these proteins under Al-stress conditions independently of SL inoculation.

### 3.4. Identification and Analysis of Specific Proteins involved in Al Response under S. luteus Inoculation in Masson Pine Seedlings

The main goal of this study was to understand how *S. luteus* helps masson pine seedlings to tolerate Al stress. So, the specific DEPs identified in LB-04 vs. LB-0 are crucial components of this mechanism. In total, we identified 198 DEPs specific to LB-04 vs. LB-0 group (Table S4). Among these 198 proteins, 79 proteins were up-regulated, and 119 proteins showed a down-regulated expression pattern in LB-04 group. A total of 136 proteins were unknown function, providing interesting novel protein resources to further investigate Al-stress response in plants. The major GO terms associated with these proteins were: biological process (gene regulation, metabolic process, oxidation and reduction process, binding process, and response to oxidative stress), molecular functions (hydrolyze activity, peptide activity, catalytic activity, and binding), and cellular components (cytosol, membrane, plastids, mitochondrion, and chloroplast).

Among the 62 proteins with known functions, 43 proteins were up-regulated, while the remaining 19 proteins showed down-regulation in LB-04 (Table S4). We further explored the known DEPs for their associated functions and GO terms and identified 17 proteins potentially associated with response to stress-induced conditions. These differential proteins were identified as chlorophyll a-b binding protein (*TRINITY_DN46586_c4_g5*), endoglucanase (*TRINITY_DN51146_c0_g2*), putative spermidine synthase (*TRINITY_DN44650_c0_g1*), NADH dehydrogenase (*TRINITY_DN48436_c0_g5*), glutathione-S-transferase (*TRINITY_DN47449_c0_g1*), LRR receptor-like protein kinase (*TRINITY_DN41128_c0_g2*), aspartic proteinase (*TRINITY_DN40732_c0_g1*), soluble starch synthase 1 (*TRINITY_DN43704_c3_g3*), T-complex protein 1 (*TRINITY_DN42434_c0_g1* and *TRINITY_DN42941_c2_g2*), purple acid phosphatase 1 (*TRINITY_DN52089_c0_g2*), NHL repeat (*TRINITY_DN47063_c0_g1*), glyceraldehyde-3-phosphate dehydrogenase (*TRINITY_DN50857_c0_g6*), SCF ubiquitin ligase (*TRINITY_DN43846_c0_g1*), ankyrin repeat domain-containing protein 2B (*TRINITY_DN44530_c4_g9*), HYL1 (*TRINITY_DN38298_c0_g1*), and AMP-dependent synthetase (*TRINITY_DN43346_c0_g2*). Identification of diverse proteins as DEPs suggests that SL-inoculation facilitates the regulation of an array of biological and molecular functions to enable masson pine to tolerate the induced Al stress.

**Figure 3:**
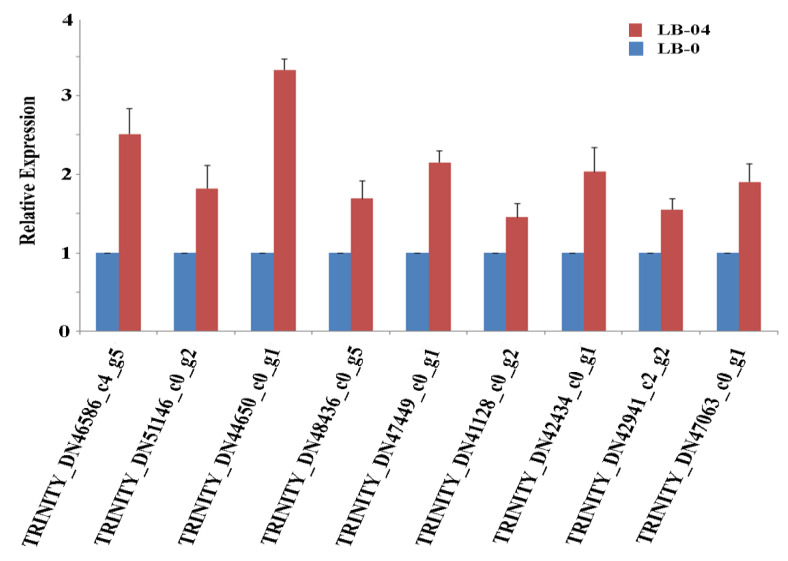
Relative expression profile of genes governing differential proteins under LB-04 (SL-inoculated Al stress condition) and LB-0 (SL inoculation condition without Al stress). Blue bars indicate gene expression levels under SL-inoculated conditions without Al treatment (LB-0), while red bars indicate gene expression levels under SL-inoculated Al stress conditions (LB-04). The error bars represent technical variations.

Next, the corresponding genes of some up-regulated proteins were identified, and their expression profiles were compared between LB-04 and LB-0 (Figure 3). All assayed genes were up-regulated under LB-04, supporting the expression pattern of their related proteins. In particular, the genes governing putative spermidine synthase (*TRINITY_DN44650_c0_g1*) and chlorophyll a-b binding protein (*TRINITY_DN46586_c4_g5*) proteins showed significantly higher expression in LB-04 than LB-0. These results emphasize that the identified genes and their corresponding proteins have a positive regulatory role in Al stress response when masson pine is inoculated with *S. luteus*. 

## 4. Discussion 

Soil acidification with an increased Aluminum (Al) toxicity level is a major reason for forest dieback [15,16]. Based on previous reports, there are two main mechanisms, viz. the exclusion mechanism and tolerance mechanism, proposed for Al stress resistance in plants. The exclusion mechanism mainly minimizes the occurrence of harmful interaction in the apoplast by hindering the entry of Al into the cytosol [56]. The symbiotic role of ectomycorrhizal fungi in *Pinus* species is also well known to naturally cope with the different biotic and abiotic stresses and increase immune response towards Al toxicity [5,44,57,58,59,60]. *S. luteus*, an ectomycorrhizal fungus, is well-known for its growth habit in adverse soil conditions, i.e., saline soils, drought, and metal toxicity [27,44,61,62,63,64,65]. *S. luteus* has been previously described to actively stimulate resistance towards high concentrations of Al by facilitating plant growth through reduced reactive oxygen species and accumulation of antioxidants [66,67,68,69,70,71]. This study aimed at uncovering the role of *S. luteus* in developing resistance towards Al toxicity in *P. massoniana* at the proteome level. 

As a result of differential expression analysis, we identified 12 core Al responsive proteins viz. chaperonin CPN60-2, Cu-Zn-superoxide dismutase precursor, drought response protein, phosphoglycerate kinase 1, and 8 unknown proteins. Abiotic stress causes enhanced protein misfolding. However, chaperones are reputed for their function in assisting protein folding under stress conditions [72,73,74]. Efficient protein repair systems and protein stability enable organisms to survive in stress conditions [75]. A study by Aremu et al. [76] described the substantial effects of chaperons on protein folding, as an adaptive strategy under Al stress. Thus, we speculated that chaperonin CPN60-2 is an important Al-responsive protein in masson pine. Another core Al-responsive protein, phosphoglycerate kinase-1, has been reported with its significant impact on plant and regulation of metabolic processes under different abiotic stress conditions [77,78]. Furthermore, Cu-Zn-superoxide dismutase precursor (CSD) gene is regulated under oxidative stress conditions [79,80] due to the downregulation of miRNA. CSD protein was up-regulated under induced Al stress conditions in this study. The antioxidant system controls oxidative cellular damage under abiotic stress conditions, specifically under Al-stress conditions [81]. Many studies have emphasized the positive regulation of oxidative stress by Al-induced genes in different plants [81,82,83,84].

Proteomics insights into induced Al stress under SL-inoculation identified 198 specific differentially expressed proteins (DEPs). GO term classified these proteins as BP: response to stress stimulus, polyamine metabolic process, cellulose catabolic process, MF: metal ion binding, chlorophyll-binding, metabolic process, and hydrolase activity, CC: photosystem I & II, and integral component of membrane. In Plants, the first steps of tolerance under stress conditions are the ability to promptly sense the stress and trigger appropriate biological responses [85]. Furthermore, stress signals and intercellular communication are vital to withstand the stress and activate the stress-related genes [85,86]. The identified DEPs include some well-known abiotic stress-responsive proteins such as chlorophyll a-b binding protein, laccase, endoglucanase, and spermidine synthase [87,88,89,90]. Differential regulation of chlorophyll a-b binding protein (*TRINITY_DN46586_c4_g5*) under LB-04 vs. LB-0 suggests its significant role in developing resistance in SL-inoculated *Pinus* against Al stress. Another protein, endoglucanase (*TRINITY_DN51146_c0_g2*), was also up-regulated under LB-04 vs. LB-0. Endoglucanase promotes cell wall development via carbohydrate binding and cellulase activity [91,92]. Remodeling of the cell wall in response to stress has been extensively studied [93,94,95,96]. The cell wall provides structural integrity, supports cell division and acts as the first defense line against stressful conditions such as Al stress [97,98]. Putative spermidine synthase protein was also identified as specific DEPs in LB-04 vs. LB0. Sang et al. [99] reported a positive effect of exogenous spermidine in tomato seedlings under abiotic stress conditions. Another report by Chen et al. [100] also suggested the involvement of spermidine in the tolerance towards abiotic stress conditions. Furthermore, NADH dehydrogenase [101,102], glutathione-S-transferase [103,104], LRR receptor-like protein kinase [105], aspartic proteinase [106,107], soluble starch synthase 1 [108,109], T-complex protein [73], purple acid phosphatase 1 [110,111], NHL repeat [112], glyceraldehyde-3-phosphate dehydrogenase [113,114], SCF ubiquitin ligase [115], ankyrin repeat domain-containing protein 2B [116], and HYL1 [117] were also identified as specific DEPs in response to Al stress under SL-inoculation in this study. These proteins have been reported for their direct or indirect regulatory roles in plant responses towards biotic and abiotic stress conditions, including Al stress. Interestingly, the expression of some of the DEPs identified in this study (i.e., chlorophyll a-b binding protein, glucanase, purple acid phosphatase) has been found regulated by methyl-jasmonate treatment in rice [118]. Likewise, Wang et al. [119] described jasmonate and aluminum crosstalk in tomato. Hence, Al sensing under *S. luteus* inoculation in masson pine might induce the activation of JA pathway. Further study is required to understand the relationship between Al stress and jasmonic acid pathways under SL inoculation in masson pine.

The up-regulation of several DEPs emphasized a network of regulatory processes to cope with the Al stress in SL-inoculated masson pine. Furthermore, the expression patterns of genes governing some of the above-mentioned proteins also confirmed the positive regulatory roles of these proteins. Our results are consistent with the various studies reporting the involvement of these genes in abiotic stress responses conditions in different plants [120,121,122,123,124,125,126]. However, we also identified several proteins, including ABI3-interacting protein 2, Tyrosine-tRNA ligase, water deficit stress-inducible protein LP3-2, serine/threonine-protein phosphatase 5, tripeptidyl-peptidase 2 isoform X1, and xyloglucan endotrans- glucosylase/hydrolase 1, which were down-regulated in LB-04 vs. LB-0. In contrast to our results, previous reports showed elevated expression of these proteins under abiotic stress conditions [17,127,128,129,130,131]. We suspect that a down-regulation of these proteins might be attributed to SL-inoculation. 

## 5. Conclusions

We reported the proteomic profile changes in *P. massoniana* attributed to Al stress and examined the effect of *S. luteus*. As a result, we identified 12 core Al responsive proteins differentially expressed between different sets of treatments. Furthermore, we identified 198 specific proteins differentially expressed under SL-inoculated Al stress conditions. Further molecular characterizations of these proteins and their corresponding genes can provide deeper insights into the mechanisms underlying Al-stress resistance in *P. massoniana* under S. *luteus* inoculation.

## Figures and Tables

**Table 1:** Specific forward and reverse primers used for qRT-PCR.

Gene ID	Forward Primer Sequence	Reverse Primer Sequence	E Value (%)	R^2^
*TRINITY_DN51949_c0_g2*	CCGTCATCGCTCCAGT	CACAGTTCGCCCTTCA	93.2	0.94
*TRINITY_DN44650_c0_g1*	GGAAAGTGGGTGGTCT	AGGAGTTCGTGGGATT	91.5	0.86
*TRINITY_DN50464_c0_g3*	ATTGATAGGAGGCTGA	TGAGGGAACTACGAGA	89.6	0.95
*TRINITY_DN39799_c0_g2*	GAGACAATGTGGTGGC	TTTGGCAGTGTAAGCA	94	0.91
*TRINITY_DN39005_c0_g1*	GCTACACCCTCGCAGT	AGCACGACCAGGAAAC	87.6	0.88
*TRINITY_DN43045_c0_g9*	CCTTGAACCCAAATACA	ACGGGCTTACCAGTCT	91.3	0.92
*TRINITY_DN43345_c2_g1*	AACAAGCCGTTGGACT	GGGAACAAAGGATGGG	92.4	0.95
*TRINITY_DN47656_c0_g2*	CCTGTATTGCCTGATG	GACGAGATGGTGGAGT	88.5	0.93
*TRINITY_DN47910_c0_g1*	TCACCTGCCATACAAA	TCCAGCATCAAAGAAA	91	0.93

## Data Availability

The mass spectrometry proteomics data have been deposited to the ProteomeXchange Consortium (http://proteomecentral.proteomexchange.org, accessed on 12 January 2021) via the iProX partner repository with the dataset identifier PXD023856.

## Supplementary Materials

The following are available online at https://www.mdpi.com/2075-1729/11/2/177/s1, Figure S1: Proteome profiling of masson pine (**a**) Protein sequence coverage distribution (**b**) Peptide Ion score distribution (**c**) Protein ratio distribution for CK-04 vs. CK-0 (**d**) Protein ratio distribution for LB-04 vs. LB-0, Table S1: List of identified proteins and their quantification, Table S2: Differential protein expressed in CK-04 Vs CK-0 with corresponding GO annotations, Table S3: Differential protein expressed in LB-04 Vs CK-04 with corresponding GO annotations, Table S4: Differential protein expressed in LB-04 Vs LB-0 with corresponding GO annotations and KEGG pathways, Table S5: Differentially expressed proteins (DEPs) conserved between CK-04 vs. CK-0 and LB-04 vs. LB-0.
