# Supplementary figures and images for: Proteomic Insight into the Symbiotic Relationship of Pinus massoniana Lamb and Suillus luteus towards Developing Al-Stress Resistance
Source: Life (Basel). 2021 Feb 23;11(2):177. doi: 10.3390/life11020177 (PMC7926926; doi:10.3390/life11020177)

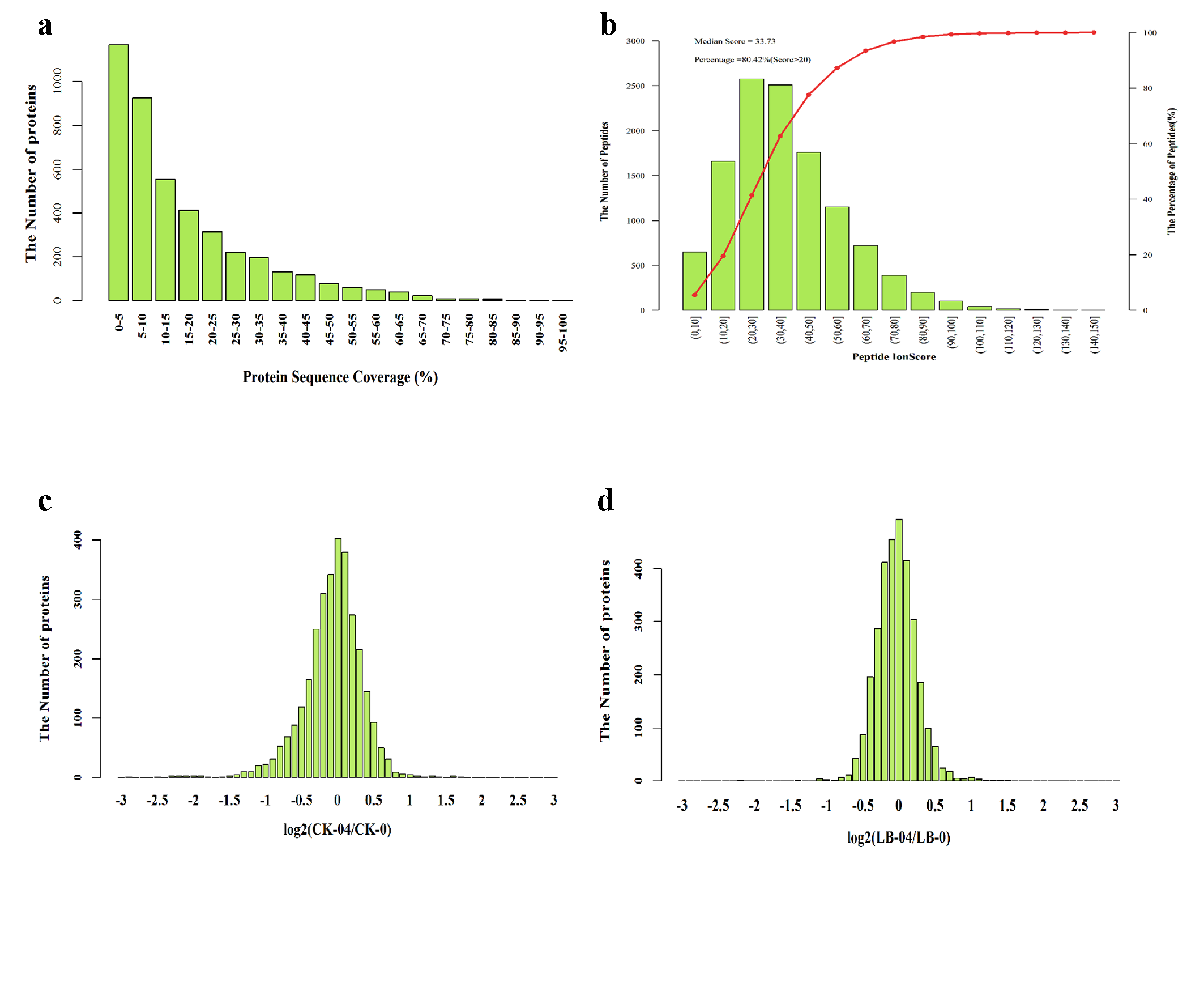

Supplement: Supplementary file 1 [file life-11-00177-s001.zip › supplementary Figure 1.tif]
